# Digital Volumetric Analysis of CAD/CAM Polymeric Materials after Tooth Brushing

**DOI:** 10.3390/polym14173499

**Published:** 2022-08-26

**Authors:** Cristian Abad-Coronel, Andrea Palomeque, Nancy Mena Córdova, Paulina Aliaga

**Affiliations:** 1 Prosthodontics Department, Faculty of Dentistry, Universidad San Francisco de Quito, Quito 170901, Ecuador; 2 Department of Digital Dentistry and CAD/CAM Materials, Faculty of Dentistry, Universidad de Cuenca, Cuenca 010107, Ecuador; 3 Faculty of Dentistry, Universidad San Francisco de Quito, Quito 170901, Ecuador

**Keywords:** CAD/CAM, composite, wear test, simulated brushing, volumetric analysis

## Abstract

The objective of the study was to evaluate the volumetric wear of four composite materials for CAD/CAM (computer-aided design/computer-aided manufacturing) systems. The materials evaluated were: Cerasmart (CER), Shofu Block HC (SBH), Tetric CAD (TEC) and Brava (BRA). All the samples (n = 40) were subjected to simulated brushing (100,000 cycles). Wear was evaluated by superimposing pre-and post-brushing scans obtained with an intraoral optical scanner (CEREC Primescan; Dentsply Sirona, Germany), which were subsequently imported into the OraCheck software 5.0 (Dentsply Sirona, Germany). The data were analyzed by ANOVA test and Tukey’s HSD test was used for multiple comparisons. Cerasmart showed the least wear after brushing. All the tested materials exhibited mass loss.

## 1. Introduction

CAD/CAM (computer-aided design/computer-aided manufacturing) technology was introduced in the 1980s, and its importance and popularity have increased rapidly, allowing clinicians to provide indirect restorations even in a single appointment [1]. Restorative materials processed using the chairside workflow are grouped into three types: (1) ceramic-based materials (glass matrix, polycrystalline); (2) plastic-based materials (polymethyl methacrylate, composite); and (3) hybrid materials that contain a combination of the previous two [2]. Chairside restorations have shown acceptable clinical results with survival rates of 97% at 5 years and 90% at 10 years [3].

Ceramic-based materials are biocompatible and have high values of hardness and wear resistance [4]; however, their abrasiveness against antagonist dentition is still a clinical challenge [5], with high rates of wear in the enamel and in restorations performed on antagonist teeth [6]. On the other hand, CAD/CAM composite plastic-based materials have a low modulus of elasticity which allows them to absorb stresses and decrease susceptibility to fracture and chipping [4]. These materials are fabricated by polymerization at high pressures and temperatures, which improves their mechanical properties, generating a more homogeneous and less porous material compared to conventional resin composites. Restorations made with these materials can be machined in less time and repaired more easily than ceramic restorations [7]. However, CAD/CAM resin composites have lower wear resistance compared to blocks of hybrid or ceramic material, which could affect their longevity [4].

Recently, nanofilled resin composite blocks have been introduced as alternatives for ceramics with faster processing characteristics, including Cerasmart^®^, which is a resin composite block consisting of a polymeric matrix reinforced by nanohybrid ceramic fillers [2,4]; Shofu Block HC^®^, a composite block consisting of an organic part composed of a triethylene glycol dimethacrylate matrix and urethane dimethacrylate and another inorganic part consisting of a densely packed nanofiller made mainly of zirconium silicate particles [8]; Tetric CAD^®^, a nanohybrid composite material consisting of a mixture of cross-linked dimethacrylates and inorganic fillers [9]; and finally, Brava^®^, which is described by its manufacturer as a reinforced composite based on methacrylic monomers and glass-ceramic fillers [10].

One of the important properties of dental materials is their wear resistance. The wear rate is defined as the loss of restorative material and/or its antagonist [11]. The volume of material lost during the interaction between two surfaces is the parameter of choice to evaluate the in vitro wear of resin-based restorative materials. According to tribology, four fundamental wear mechanisms can occur: abrasion, adhesion, fatigue and corrosion [12]. The wear of teeth and restorations in the oral cavity is a complex multifactorial process. In this aspect, tooth brushing produces abrasion on polymeric materials via the bristles of the toothbrush and toothpaste through a mechanical process independent of occlusion [13], causing stress on the organic matrix, the fillings and their interfaces, which influences their resistance. As the fillers are incorporated into the organic matrix through a chemical treatment of their surfaces, this interface undergoes stresses and detaches from the matrix showing different patterns [14].

Several quantitative analysis methods have been described to measure the in vitro wear of dental materials, including measurement of surface roughness of worn specimens, differences in thickness of specimens before and after wear, and the weight loss of worn specimens [15]. It is important to assess the wear of dental materials because of the consequences to occlusal surfaces, loss of vertical dimension, changes in the functional path of masticatory movements, fatigue of masticatory muscles, esthetic defects, and bacterial plaque retention [14].

The objective of this in vitro study was to evaluate the volumetric wear of four CAD/CAM composite materials after simulated tooth brushing using a three-body wear test. The null hypothesis of this in vitro study was that the CAD/CAM materials investigated would not show significant differences in terms of volumetric wear after tooth brushing.

## 2. Materials and Methods

In this study, 4 different types of CAD/CAM composite materials were evaluated: Cerasmart (GC), Tetric CAD (Ivoclar Vivadent), Shofu Block HC (Shofu), and Brava (FGM). Information about the materials investigated is presented in Table 1.

### 2.1. Sample Preparation

A scan was made of a printed model of an upper jaw, with a central incisor prepared in chamfer with a thickness of 1.2 mm for a vestibular veneer. The restoration was designed using CEREC 5.1.3 software. The design parameters were standardized for all samples, with a minimum thickness of 1 mm. Subsequently, the restoration was milled on the MCXL unit (Dentsply Sirona, Germany). Ten samples were fabricated for each group (n = 10). The samples were subjected to specular polishing using a plush and polishing paste (AP Esthetic, Dusseldorf, Germany) for 60 s. They were washed with water and dried with compressed air for 20 s.

### 2.2. Wear Test

**Figure 1:**
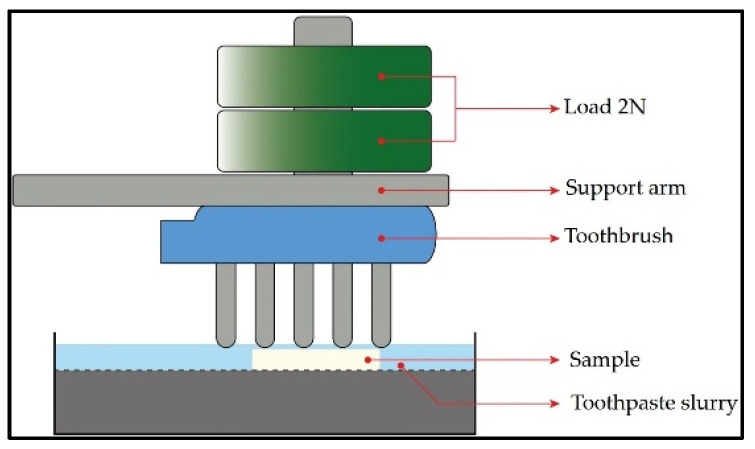
Representative image of the brushing tests.

Wear was evaluated by applying the tooth brushing test using automatic tooth brushing equipment (MEV 3T-10XY; Odeme Dental Research, Luzerna, Brazil). Hard bristle nylon brushes (Colgate Extra clean; Colgate–Palmolive, Bogotá, Colombia) were used on each brush head under a load of 2 N in a direction perpendicular to the sliding surface. Brushing was performed at a frequency of 1.2 Hz for a total of 100,000 cycles. A suspension of 150 g of toothpaste (Colgate Total 12; Colgate–Palmolive, Colombia) was injected with 1 L of distilled water every 5000 cycles to ensure that the surface remained moist. The mixture was stirred evenly before each addition to prevent particles in the toothpaste from settling to the bottom. The test was carried out at room temperature. At the end of the brushing test, each sample was cleaned with running water and dried with compressed air. (Figure 1).

### 2.3. Assessment of Volumetric Wear

The volumetric wear of the materials was measured by superimposing pre- and post-brushing test scans obtained with an intraoral optical scanner (CEREC Primescan; Dentsply Sirona, Bensheim, Germany). The data acquired from the digitization of each sample were imported in STL format into the OraCheck software 5.0 (Dentsply Sirona, Germany), which allows for a 3D comparison between two or more digital scans using the best-fit algorithm.

### 2.4. Statistical Analysis

The data records for each group of samples were compiled in an Excel file (version 16, Microsoft, Redmond, WA, USA) and imported into the SPSS program (IBM version 26 in Spanish, Madrid, Spain). The ANOVA test was used to test the hypotheses of the research to determine if there were significant differences in wear according to the materials involved in the experimental design. The HSD Tukey test was used as the statistic for the multiple comparisons test.

## 3. Results

### Wear Test

**Figure 2:**
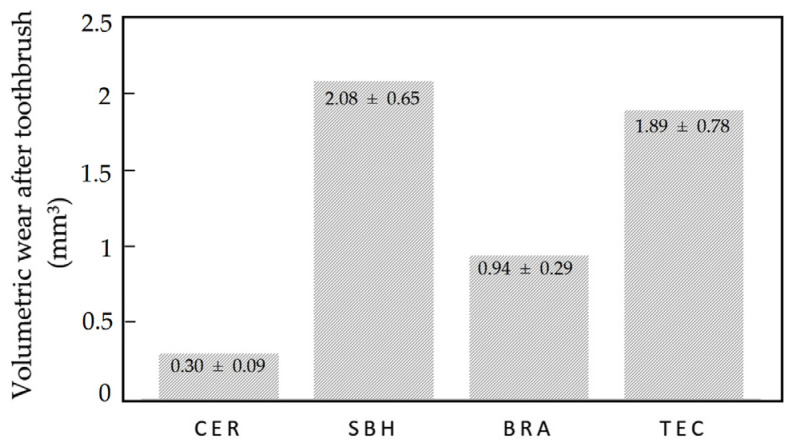
Graph of average volumetric wear for each material.

**Figure 3:**
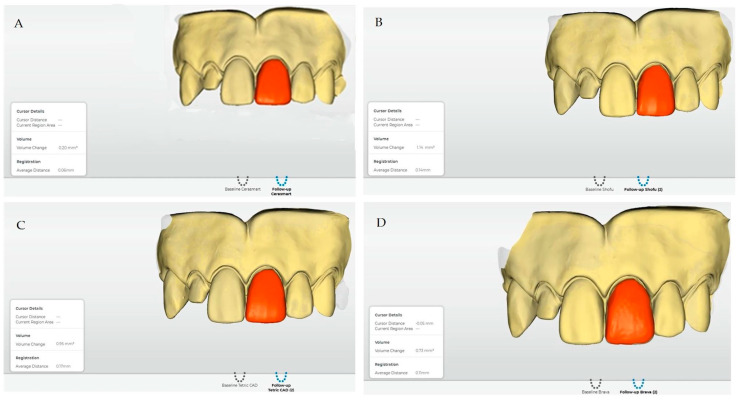
Result of the initial scan and post-wear scan overlay in the Oracheck software 5.0: (**A**) Cerasmart (CER); (**B**) Shofu Block HC (SBH); (**C**) Tetric CAD (TEC); (**D**) Brava (BRA).

After the experiment, the mean values and standard deviations of volume changes of the materials are shown in Table 2. SBH (2.08 ± 0.65 mm^3^) showed the greatest amount of wear, followed in decreasing order by TEC (1.89 ± 0.78 mm^3^), BRA (0.94 ± 0.29 mm^3^) and CER (0.30 ± 0.09 mm^3^). Figure 2 shows the differences between the averages obtained for the volumetric wear for each material; it was observed that CER is the lowest and SBH the highest. Regarding the variability of the materials, BRA presented the greatest coefficient of variation with 41.0%, i.e., the measurements for this material showed greater variability with respect to the average wear; for the other three materials, the coefficient of variation was 31%. Examples of measurements made with the Orachek software 5.0 are shown in Figure 3.

Using an ANOVA test, it was determined that there were significant differences in wear between the polymeric materials evaluated (*p*-value = 0.001 < 0.05). Table 3 lists the HSD Turkey statistic, showed significant differences in wear between CER with SBH (*p*-value = 0.003 < 0.05); and CER with TEC (*p*-value = 0.002 < 0.05). The material that obtained lower average wear values was CER (0.30 ± 0.09 mm^3^) followed by BRA material (0.94 ± 0.29 mm^3^), and they are a homogeneous group in terms of their similar average wear characteristics.

## 4. Discussion

In any restoration, surface quality is an important factor to determine clinical success. Ideally, it should be stable over its expected lifetime. However, the surface’s appearance changes due to wear caused by the oral environment and other external factors. Toothbrushing is one such factor. Abrasive particles in toothpastes can increase surface roughness and decrease gloss, affecting the esthetic quality and longevity of a restoration [17]. Clinical studies allow for the ideal conditions for establishing tooth wear, but require more time and patient commitment for follow-up and do not have control over important variables such as masticatory forces and environmental factors [18]. While in vitro studies can be difficult to interpret in clinical practice, they offer controllable conditions to achieve experimental objectives [19]. Regarding the wear resistance, we used toothbrushing to evaluate the resistance of materials. The samples were submitted to 100,000 brushing strokes, which simulated 10 years of clinical wear [20].

For the evaluation of in vitro wear of dental materials, several quantitative analysis methods have been described, among which can be mentioned the measurement of the roughness of the worn surface (Ra) [1,21,22], the difference in thickness of the samples before and after wear [23], and the weight loss of the worn samples [24]. Hartkamp et al. compared volumetric wear measurement using optical profilometry vs. an intraoral scanner and concluded that intraoral scanner-based measurement is a cost-effective, fast and easy-to-apply tool with acceptable reliability compared to profilometry that requires a model to perform the analysis [25]. In another study by Choi et al., the in vitro wear of dental materials was evaluated, eliminating the errors associated with the use of replicas because volume losses were measured directly on antagonists and material specimens, rather than indirect techniques using cast replicas. Additionally, 3D wear was measured using a non-contact scanner, which has been shown to be more effective and accurate, allowing objective data on material wear to be obtained. [15]. In the present study, four CAD/CAM composite materials currently used in prosthodontics were compared. An intraoral scanner was used to collect pre- and post-simulated brushing test data and subsequently evaluated in OraCheck software to determine the volume wear of each material.

The results of this study showed that wear was influenced by the type of material and simulated toothbrushing. Thus, the null hypothesis was rejected. The four blocks evaluated had different amount of inorganic content, as shown in Table 1. This can be expressed from the highest to lowest amount as: TEC > CER > SBH > BRA. The results showed that the material that obtained the lowest average wear was CER, followed by BRA material. TEC and SHB materials exhibited the higher wear. The wear resistance of composite materials depends on several factors, such as the type of filler content, the interfacial bond between the filler and the matrix, the degree of polymerization of the resin matrix, and the manufacturing process [1,26]. Although the inorganic filler content of TEC is higher than CER, its wear was higher than CER. According to manufacturer specifications, TEC has larger polygonal glass particles in its composition, whereas CER has well-distributed spherical nano-sized filler particles. The high filler loading, and small-sized filler particles result in a smaller interparticle distance, which could contribute to the better resistance to wear by toothbrushing for the CER material. In the case of SBH, which consists of large silica and zirconium silicate filler particles of spherical shape with less filler content, it is easily worn out by toothbrushing [1,22]. Oouchi et al., showed in their study that wear resistance is affected by filler content and by particle size, shape and distribution, with high filler content and small particles being more advantageous [27] Koizumi et al. tested different “resin-ceramic” CAD/CAM materials after simulating a toothbrushing of five years. The results reported higher wear after toothbrush abrasion on SBH samples compared to CER, with a decrease in their surface quality being observed with resin loss and the appearance of spherical fillers. They attribute this finding to the fact that the SBH filler particles are harder than the surrounding resin matrix, so they would wear more easily during tooth brushing [28]. In the present study, SBH exhibited higher wear compared to the other composite materials. In another study, Schmitt de Andrade et al. evaluated the effect of simulated tooth brushing on the surface wear of CAD/CAM materials, in which those materials with glass matrix had lower wear values than composite resins [29]. This result was related to evidence from other studies that materials with higher hardness are less prone to abrasive wear [13,30,31,32]. Hardness is defined as a measure of resistance to indentation and indicates the ease of finishing and scratch resistance [33]. It should also be noted that hardness is not the only predictor of material wear. Composite resin wear will not only result from friction but will also occur due to chemical degradation caused by an aggressive environment in the oral cavity [34].

The most common monomers used in dental resins are triethylene glycol dimethacrylate (TEGDMA), urethane dimethacrylate (UDMA), bisphenol A glycol dimethacrylate (Bis GMA), and 2-hydroxyethyl methacrylate (HEMA) [35]. It has been observed that composites with TEGDMA absorb more water and create a more flexible polymer network, while those containing UDMA and Bis-EMA absorb less water and create stiffer networks. Bis-GMA leads to the formation of the stiffer network, which absorbs less water than the resin made by TEGDMA, but more than the resins made by UDMA and Bis-EMA [36]. Şen et al. evaluated the effects of immersion in food-simulating liquids and tooth brushing on the surface of dental materials, whose association between these chemical and mechanical processes have shown clinical relevance, presenting chemical degradation on Bis-GMA molecules [37]. In another study in which surface changes in composite materials were evaluated in relation to brushing time, it was concluded that a brushing time of 10 h was required to evaluate the potential for deterioration [38]. In the present study, during the simulated brushing, the specimens were exposed to a solution of distilled water and dentifrice at room temperature for a time of 10 h (100,000 cycles) (Figure 1), so it could be hypothesized that there was higher wear of the materials that presented Bis-GMA and TEGDMA in their composition, corresponding to SHB and TEC. This relationship between the higher volume loss in the wear of materials that presented TEGDMA in their composition was reported in another study [39]. Moreover, in the case of SHB, it can be hypothesized that the absorbed water would cause hydrolysis of the interfacial silane coupling agent, especially in the case of zirconium silicate which does not silanize effectively due to its high crystalline content [40]. The information provided by the BRA manufacturer on the composition of the CAD/CAM composite is so modest that, in this respect, no comparisons can be made.

Supposedly, the type of toothbrush and the stiffness of the bristles have hardly any effect on the wear of the resin composite [41]. In this study, a toothbrush with stiff bristles was arbitrarily selected. The influence of the toothbrush on abrasiveness is negligible when water is used as a substrate, but when a toothpaste is added, the influence of the brush is of great importance, where a softer toothbrush can cause similar or even greater abrasion than one with stiffer bristles [42]. In this study, toothbrushing was performed with dentifrice with a relative dentin abrasiveness (RDA) level of 70, which is considered a low abrasion value [43], diluted in distilled water, which is very different from this dilution occurring in saliva in the oral cavity. The use of saliva as a lubricant tends to reduce the rate of wear compared to water. Therefore, friction and abrasion between moving surfaces may be overestimated [44].

Among the limitations of the present study, the effects of occlusal loads, pH, and temperature changes occurring in the oral environment were not considered. Further studies are needed to mimic the real situation of a composite restoration in the oral cavity as far as possible, and to further investigate the factors that cause composite deterioration in the future, providing more reliable information for clinical practice.

## 5. Conclusions

Within the limits of this study, in the comparison of the four different groups of restorative materials, all materials evaluated showed varying degrees of volume loss. In addition, the wear resistance was related to the filler content. After brushing, the material with the least wear was Cerasmart, information that may be useful in predicting the performance of these materials in the restorative clinic for the anterior sector.

## Figures and Tables

**Table 1:** Technical characteristics of the CAD/CAM materials evaluated in this study.

Material	Code	Manufacturer	Composition	% Mass	Lot Number
**Cerasmart**	CER	GC Corp., Tokyo, Japan	Bis-MEPP, UDMA, DMA, Silica (20 nm), barium glass (300 nm) [16]	71	2010121
**Shofu Block HC**	SBH	Shofu Inc, Kyoto, Japan	UDMA, TEGDMA, sílica powder, barium glass, micro-fumed silica, zirconium silicate [16]	61	1116204
**Tetric CAD**	TEC	Ivoclar Vivadent, Liechtenstein	Bis-GMA, Bis-EMA, TEGDMA, UDMA, barium glass (<1 µm), silicium dioxide (<20 nm) [9]	71	X27858
**Brava**	BRA	FGM, Joinville, Brazil	Organic matrix, inhibitor, initiator and stabilizer, Glass-ceramic fillers, silica and pigments. (40 nm a 5 µm) [10]	58	110121

**Abbr.:** Bis-MEPP: 2,2-Bis(4-methacryloxypolyethoxyphenyl) propane; UDMA: urethane dimethacrylate; DMA: dimethacrylate; TEGDMA: triethylene glycol dimethacrylate; Bis-GMA: bisphenol A diglycidylether methacrylate; Bis-EMA: ethoxylated bisphenol-A dimethacrylate.

**Table 2:** Means, standard deviations (SDs) and variation coefficient of volumetric losses of materials after wear.

Material	n	Mean ± SD (mm^3^)	Min	Max	Variation Coefficient
CER	10	0.30 ± 0.09	0.20	0.41	31.9%
SBH	10	2.08 ± 0.65	1.14	2.93	31.1%
BRA	10	0.94 ± 0.29	0.74	1.27	30.3%
TEC	10	1.89 ± 0.78	0.16	2.55	41.0%

**Table 3:** Results of the multiple comparisons test with Tukey HSD.

(I) Group	(J)	Mean Difference (I-J)	*p*-Value
CER	SBH	−1.78 *	0.003
	BRA	−0.65	0.554
	TEC	−1.59 *	0.002

Note: * Mean difference is significant at the 0.05 level. HSD Tukey statistic. Dependent variable is the wear resulting from the simulation with abrasive brushing.

## Data Availability

https://drive.google.com/drive/folders/1PI2XUPaY9K6_E-4KtMgtUK2sQxBE6i6C?usp=sharing.
